# Macronutrient Analysis of Target-Pooled Donor Breast Milk and Corresponding Growth in Very Low Birth Weight Infants

**DOI:** 10.3390/nu11081884

**Published:** 2019-08-13

**Authors:** Ting Ting Fu, Paige E. Schroder, Brenda B. Poindexter

**Affiliations:** 1 Perinatal Institute, Division of Neonatology, Cincinnati Children’s Hospital Medical Center, Cincinnati, OH 45229, USA; 2 Department of Pediatrics, University of Cincinnati College of Medicine, Cincinnati, OH 45267, USA

**Keywords:** donor breast milk, human milk, milk analysis, very low birth weight, preterm, growth

## Abstract

The macronutrient composition of target-pooled donor breast milk (DBM) (milk combined strategically to provide 20 kcal/oz) and growth patterns of preterm infants receiving it have not been characterized. Caloric target-pooled DBM samples were analyzed by near-infrared spectroscopy. Weekly growth velocities and anthropometric z-scores were calculated for the first 30 days and at 36 weeks corrected gestational age (CGA) for 69 very low birthweight (VLBW) infants receiving minimum one week of DBM. Samples contained mean 18.70 kcal/oz, 0.91 g/dL protein, 3.11 g/dL fat, 7.71 g/dL carbohydrate (*n* = 96), less than labeled values by 2.43 kcal/oz and 0.11 g/dL protein (*p* < 0.001). By week 3, growth reached 16.58 g/kg/day, 0.95 cm/week (length), and 1.01 cm/week (head circumference). Infants receiving <50% vs. >50% DBM had similar growth, but infants receiving >50% DBM were more likely to receive fortification >24 kcal/oz (83% vs. 51.9% in the <50% DBM group; *p* = 0.005). From birth to 36 weeks CGA (*n* = 60), there was a negative z-score change across all parameters with the greatest in length (−1.01). Thus, target-pooling does not meet recommended protein intake for VLBW infants. Infants fed target-pooled DBM still demonstrate a disproportionate negative change in length z-score over time.

## 1. Introduction

Neonatal practitioners commonly assume that human breast milk contains 20 kcal/oz, but the macronutrient content of human milk depends on many factors including gestational age [1]. Compared to preterm milk, term milk has less energy and protein, and in both populations, energy and protein content decreases over time as lactation progresses [2]. Because donor breast milk (DBM) commonly comes from mothers of term babies later in lactation, one major concern regarding the use of DBM in preterm infants is its nutritional adequacy, particularly its protein concentration, since higher protein intake and increased linear growth are associated with improved neurodevelopmental outcomes [3,4,5]. Pooled DBM has been shown to contain as low as 14.6 kcal/oz [6], and protein content in maternal preterm milk ranges from 1.2 to 1.7 g/dL in the first four weeks of lactation whereas the content in DBM is generally accepted as 0.9 g/dL [7]. For infants weighing less than 1 kg, the European Society for Pediatric Gastroenterology Hepatology and Nutrition (ESPGHAN) recommends an enteral intake of 4.0–4.5 g/kg/day of protein [8], which correlates to 2.7–3.0 g/dL assuming enteral fluid intake of 150 ml/kg/day. However, protein fortification for human milk is difficult, and standard multicomponent human milk fortifiers may still be insufficient as manufacturers of commercially available bovine-derived human milk fortifiers assume a baseline protein concentration of 1.4–1.6 g/dL in human milk [9,10].

Earlier studies comparing fortified DBM versus premature infant formula reported notably decreased growth velocities in the DBM group, particularly in weight and length [11,12]. However, more recent studies have shown similar growth can be achieved with adequate monitoring and fortification [13,14,15].

Target-pooling is a method that some milk banks employ to increase nutritional content by combining milk of multiple donors strategically, rather than randomly. One specific technique is to add skimmed fat from lower-calorie breast milk to higher-calorie breast milk to mimic hind milk and achieve a minimum of 20 kcal/oz. However, it is unclear how protein concentrations of DBM and subsequent infant growth are affected by caloric targeting. The objective of this study was to characterize the macronutrient composition and variability of caloric target-pooled DBM and the corresponding growth velocities of very low birth weight (VLBW) infants who receive it.

## 2. Materials and Methods 

### 2.1. Patient Sample 

This prospective observational study was performed at the neonatal intensive care unit (NICU) at TriHealth Good Samaritan Hospital in Cincinnati, Ohio, with milk analysis conducted at Cincinnati Children’s Hospital Medical Center (CCHMC). The study was approved by the Institutional Review Board at both institutions with waiver of informed consent (CCHMC 2015-5191, TriHealth 15-085).

VLBW infants admitted to the NICU from December 2015 to April 2017 who received more than 1 week of DBM during the first 30 days of life as supplementation to maternal milk were eligible. Infants who transferred to another hospital or passed away in the first 30 days of life or did not follow the standardized feeding protocol (see below, Section 2.3) were excluded.

### 2.2. Milk Collection and Analysis

During the time period in which eligible infants were admitted, target-pooled DBM purchased from Mothers’ Milk Bank of Ohio (MMBO, Columbus, Ohio) were screened for unique pools, and a representative bottle for each unique pool was marked. Caloric and protein content, as measured by MMBO and labeled on each bottle, was recorded. Per unit protocol, NICU milk technicians prepared feedings from refrigerator-thawed bottles by hand homogenizing and either pipetting or pouring into measured containers. From each marked bottle, a minimum of 1 ml of the remaining milk, more if allowed, was saved, and kept frozen for sample collection. For analysis, samples were heated to 37 °C, gently homogenized by hand, then homogenized for 30 seconds using a sonicator. Using a near-infrared (NIR) human milk analyzer (SpectraStar 2400, Unity Scientific, Brookfield, Connecticut), which was calibrated using a bias set of human breast milk obtained from MMBO, samples were then analyzed in 1–1.5 mL aliquots, triplicate if volume allowed.

### 2.3. Standardized VLBW Feeding Protocol

DBM is utilized for infants with birth weight </= 1500 g when maternal milk is not available for the first 30 days of life. Enteral feedings are initiated within 48 h of birth at 15 mL/kg/day for 3 days and subsequently advanced by 10 mL/kg/day every 12 h to a goal of 160 mL/kg/day. Fortification to 24 kcal/oz occurs at 75 mL/kg/day, usually day of life 7, using Similac (Abbott Nutrition, Columbus Ohio) human milk fortifier hydrolyzed protein concentrated liquid (HMF-HPCL). Additional fortification occurs as clinically indicated for poor growth using additional HMF-HPCL, Similac Special Care 30, Similac NeoSure, and/or Similac Liquid Protein Fortifier, per dietitian’s discretion. In addition to enteral intake, parenteral nutrition provides 2.5 g/kg/day of protein starting on day of life 1, then 3.5 g/kg/day onward until intake is limited by fluid volume. 

### 2.4. Enteral Intake Data

Enteral intake data were obtained from charted enteral feeding volumes and fortification status of donor and maternal milk for the first 30 days of life or until DBM was transitioned to formula. Percentage of DBM intake was calculated by dividing the volume of DBM by the total volume of human milk that the infant received during the studied time period. The last day on which DBM was given, whether the infant was still receiving DBM on day 30, and the highest caloric density of fortification of DBM were recorded. Utilizing the NICU’s established milk tracking system (Women and Infants, Timeless Medical Systems, Charlottetown, Prince Edward Island, Canada), the source pool from each bottle of DBM that the infant received was identified.

### 2.5. Anthropometric Data

Weekly weight, length, and head circumference (HC), as recorded by clinical care, were collected until 4 weeks of age and also at 36 weeks corrected gestational age (CGA). Growth velocities, Olsen body mass index (BMI) [16], and Fenton z-scores [17] were calculated for each time point. Weight velocity was calculated using the two-point model. Per unit practice, length boards were used as needed to verify measurements that appeared abnormal. Outliers in length and HC (a gain of greater than 3 cm or a loss of greater than 2 cm) were excluded. For patients discharged prior to 36 weeks CGA, measurements at 35 weeks CGA were recorded if available. Small for gestational age (SGA) was defined as a birth weight below the 10th percentile, and appropriate for gestational age (AGA) was defined as birth weight between the 10th and 90th percentile.

### 2.6. Statistical Analysis

Analyses were performed using SAS Studio version 3.71. NIR results were compared to labeled values using paired t-test analysis. Subgroups comparisons of SGA vs. AGA status and DBM intake percentage (<50% vs. >50%) were analyzed using 2-tailed 2-sample t-tests and χ^2^ test. *P* < 0.05 was considered statistically significant. A sample size of 45 patients was estimated to detect a difference of 2.18 g/kg/day difference in weight (assuming full enteral feeding volume of 160 ml/kg/day with DBM fortified to 24 kcal/oz and baseline protein concentration 0.9 g/dL, yielding a projected protein intake of 3.87 g/kg/day, 0.63 g/kg/day less than ESPGHAN recommendations) with 80% power and alpha 0.05 and based on the largest randomized trial known at the time of study design describing growth in infants fed DBM [11].

## 3. Results

### 3.1. Study Infants

**Figure 1:**
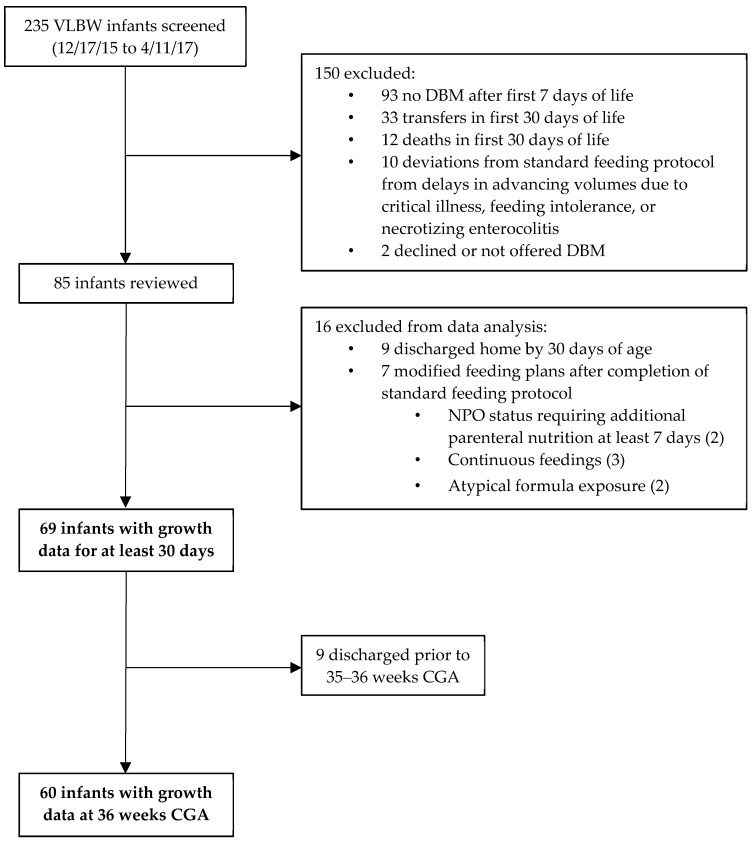
Flow diagram of study infants.

Of 235 infants screened, 85 met inclusion criteria (Figure 1). An additional 16 were excluded due to early discharge or modified feeding plans after completion of the standard feeding protocol. Thus, 69 had growth data available at 30 days, and 60 had measurements available at 36 weeks CGA. The summary of their characteristics can be found in Table 1. Of those 69 patients who had growth data, 65.9% were still receiving DBM as part or all of their feedings at 30 days old, and 5 additional patients were transitioned early from DBM to formula due to poor growth at 27–28 days. Further, 15.9% were SGA and 71.0% received increased fortification, which occurred on average at day 18.5.

### 3.2. Donor Milk Analysis

**Figure 2:**
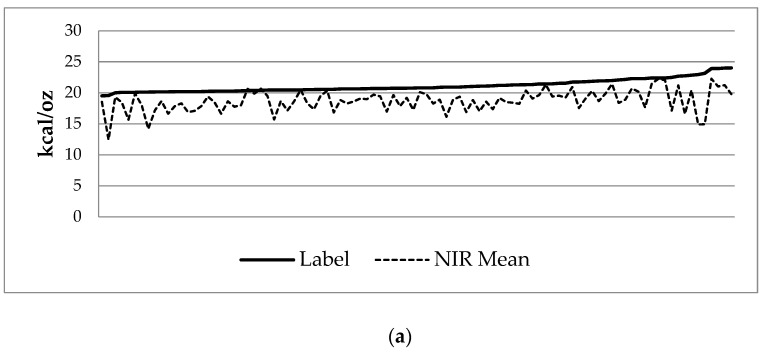

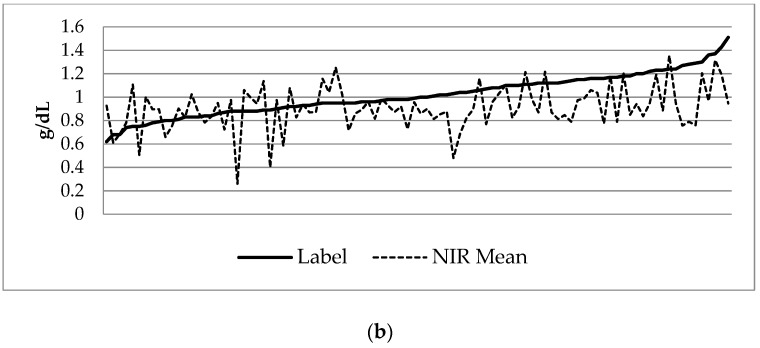
Labeled (solid line) vs. mean NIR analysis (dashed line) of (**a**) caloric and (**b**) protein content, ordered by increasing labeled values.

Samples from 96 unique pools were obtained. Review of enteral intake charting and milk tracking showed 146 unique pools of DBM were actually utilized during the study period. NIR analysis found mean contents of 18.70 ± 1.75 kcal/oz, 0.91 ± 0.19 g/dL protein, 3.11 ± 0.57 g/dL fat, and 7.71 ± 0.38 g/dL carbohydrate (Table 2). Mean coefficients of variation of triplicate or duplicate analysis were 1.61% for calories, 6.81% for protein, 2.68% for fat, and 1.81% for carbohydrate. Labeled nutritional information demonstrated mean calorie content of 21.13 ± 1.01 kcal/oz and mean protein content 1.02 ± 0.18 g/dL. On average, compared to labeled values, the samples had 2.43 kcal/oz less (*p* < 0.001) and 0.11 g/dL less protein (*p* < 0.001) (Table 3, Figure 2).

### 3.3. Growth Analysis

Mean weight velocity reached 16.58 g/kg/day by week 3, mean length velocity ranged from 0.95 to 1.03 cm/week during weeks 2-4, and mean HC velocity reached 1.01 cm/week by week 3 (Table 4). When comparing the subgroups of SGA and AGA infants, the mean velocities were statistically different for weight velocity in weeks 1 and 2 (*p* = 0.001, *p* = 0.009) (Table 4). There were no large-for-gestational-age infants. Infants whose enteral intake comprised of less than 50% DBM had similar growth velocities compared to those whose enteral intake was greater than 50% DBM with the exception of weight velocity at week 2 (*p* = 0.024) (Table 4). Further, 51.9% in the <50% DBM group and 83.3% in the >50% DBM group received fortification beyond 24 kcal/oz (*p* = 0.005).

**Figure 3:**
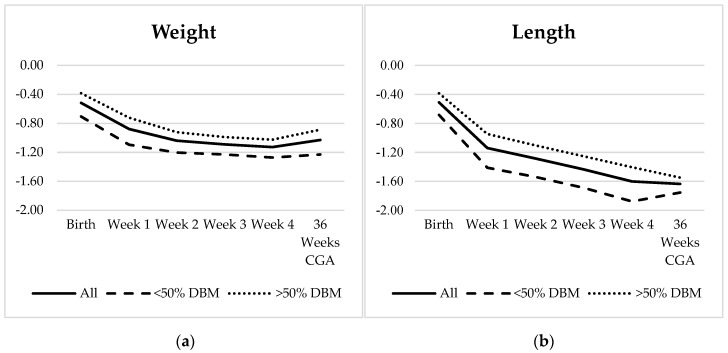

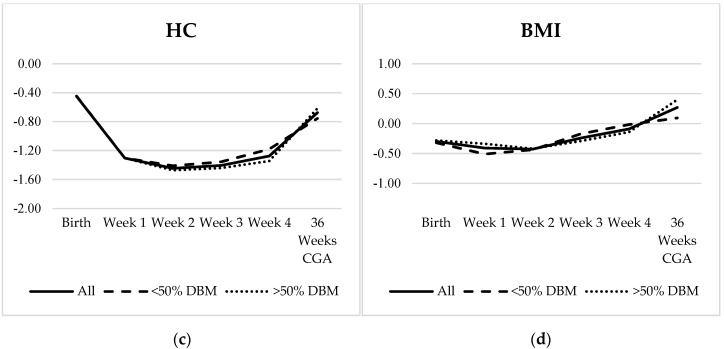
(**a**–**c**) Fenton z-score trajectories for weight, length, and head circumference (HC) and (**d**) Olsen z-score trajectory for body mass index (BMI) over time for all infants (solid line, *n* = 60), infants with <50% DBM intake (dashed line, *n* = 25), and infants with >50% DBM intake (dotted line, *n* = 35).

**Figure 4:**
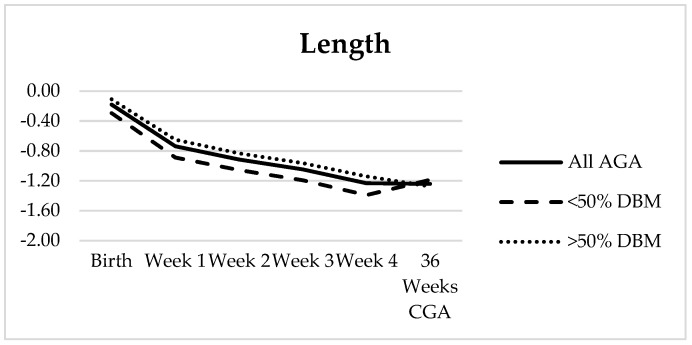
Fenton z-score trajectory for length in all appropriate-for-gestational-age (AGA) infants (solid line, *n* = 49) and subgroups of <50% DBM intake (dashed line, *n* = 18) and >50% DBM intake (dotted line, *n* = 31). Average rate of change from week 4 to 36 weeks CGA in the two subgroups were 0.12 and −0.14, respectively (*p* = 0.023).

For the 60 infants who had growth measurements available at 36 weeks CGA, the Fenton z-score decreased for HC during weeks 1–2 and for both weight and length during all four weeks. From birth to 36 weeks CGA, there was a negative z-score change across all three parameters with the greatest change seen in length (−1.01) (Table 5, Figure 3). HC z-score improved to within 0.23 of birth, and a small increase (0.1) was noted in weight z-score between week 4 and 36 weeks CGA. Olsen BMI was the only measure to have a net increase in z-score over time. Further, 11/60 infants were SGA at birth; an additional 10 AGA infants became <10% for weight by 36 weeks CGA. There appeared to be a difference between the two DBM subgroups in both weight and length, though it was not statistically significant for any measurement (Figure 3), and with the exception of the change in BMI from week 4 to 36 weeks CGA, the weekly change in z-score and also net change from birth to 36 weeks CGA were not statistically different. After excluding the 11 SGA patients, again there was no statistically significant difference between the two DBM groups except between week 4 and 36 weeks CGA where the length z-score continued to decrease by −0.14 in the >50% DBM group but increased by 0.12 in the <50% DBM group (*p* = 0.023) (Figure 4).

## 4. Discussion

NIR analysis revealed that the target-pooled DBM samples contained similar calories (18.7 vs. 18.0–18.7 kcal/oz) and protein concentrations (0.91 vs. 0.88–1.0 g/dL) compared to other recent analyses of multi-donor random-pooled DBM [18,19]. However, in these studies, samples were measured pre-pasteurization. MMBO’s labeled pre-pasteurization measurements showed the calorically targeted-pools contained mean 21.13 kcal/oz and 1.02 g/dL, reflective of their particular technique designed to mimic hind milk. As there are no dedicated regulations currently in place regarding pooling, the techniques utilized by other banks, which may include protein targeting, could result in different macronutrient ratios.

Furthermore, the measured concentrations for calories were, across the board, less than indicated on the corresponding labels (Figure 2), with one sample as low as 12.43 kcal/oz. Given that our NIR analyzer was calibrated utilizing milk and measurements provided by MMBO and that samples were collected after feeding preparations were completed for each shift, this suggests that nutrient loss likely occurred during preparation and handling. Handling from freezing and thawing of human milk has been shown to decrease caloric delivery [20], likely secondary to increased contact with plastic surfaces to which fat adheres, and the steps of feeding preparation, such as hand-homogenization and pouring versus using a transfer pipette, may also yield uneven distribution of macronutrients due to technician variation. The NIR-measured protein content was inconsistently matched with its label counterpart (Figure 2), potentially due to poor homogenization and compartmentalization. This carries implications in unequal delivery of nutrients between patients and also between feedings to individual patients, leading to unintended under- or over-nutrition. Developing consistent feeding preparation techniques to improve homogenization, minimize fat loss, and optimize nutrient delivery is an important focus for further research and quality improvement.

Growth parameters reached or approached goal velocities (15 g/kg/day for weight, 1 cm/week for length and HC) by weeks 3–4, but 71.0% of patients received additional fortification to maintain adequate growth (Table 1 and Table 4). The clinical significance of the early weight velocity in SGA patients is unclear given the small subgroup size, though it could reflect a response to metabolic programming or a larger proportion of SGA infants in the <50% DBM group, though the latter was not statistically significant. The difference in weight velocity between the two DBM intake groups at week 2 and the narrowed gap at week 3 correlates, respectively, with infants approaching full enteral volumes with little or no parenteral nutrition and the point at which increased fortification occurred, supporting previous findings that acceptable growth velocities can be achieved on a DBM diet with appropriate fortification [14].

In addition, weekly Fenton z-scores suffered, and patients did not return to birth z-scores by 36 weeks CGA (Table 5, Figure 3). The greatest z-score change was seen in length, and the Olsen BMI z-score increased correspondingly. This suggests that monitoring z-scores in addition to growth velocities is necessary to determine whether weekly growth is adequate. Furthermore, despite the controlled caloric intake provided by target-pooled DBM, standard fortification to 24 kcal/oz alone does not provide adequate nutrition. Standard fortification of DBM increases the concentration from 0.9 g/dL to 2.42 g/dL, still below recommendations. Moreover, the switch to preterm formula at 30 days could not overcome the early growth faltering on DBM, highlighted by the 10/49 (20%) of AGA infants who developed postnatal growth failure (weight <10% at 36 weeks CGA). With studies associating poor linear growth and protein intake with worse neurodevelopmental outcomes [3,5], the persistent decreasing length z-score over time is particularly concerning. Thus, this population may benefit from earlier aggressive fortification of DBM with focused targeting of protein intake before growth faltering is demonstrated.

Though there appears to be a difference in weight and length at birth between the DBM subgroups, both groups actually had similar z-score trajectories over time. This is likely due to the increased percentage of infants who received additional fortification in the group that received >50% DBM. Colaizy et al. previously noted a net change in weight z-score from birth to discharge of −0.84 in infants who received >75% DBM [21]. In our >50% DBM subgroup, 32/35 patients received >75% DBM, and the net change in weight z-score was −0.49, an improvement possibly attributable to the target-pooling. Our net z-score changes for weight and length were also similar to findings of the DoMINO trial, the largest randomized controlled trial to date comparing DBM versus preterm formula as primary diet [13]. However, despite the improved growth potential that target-pooling may offer, the negative trends remain worrisome. Interestingly, over 95% of the study milk from the DoMINO trial was also purchased from MMBO. Providers may wish to inquire what pooling technique is utilized by the milk bank that provides their unit’s donor milk, which may be different in content than the donor milk utilized in our study and the DoMINO study, thus limiting the generalizability of these findings.

Additionally, there was a gain in length z-score between week 4 and 36 weeks CGA for those who received <50% DBM and a decrease for those who received >50% DBM, though it was only statistically significant once the SGA infants were removed (Figure 4). Both of these groups transitioned to preterm formula as backup at 30 days, though many infants in the former likely continued to receive a larger percentage of maternal milk. Further investigation into the later feeding characteristics of these two cohorts and also comparison to infants who received almost exclusive maternal milk may provide additional insight.

One limitation of this study is the irregular sampling bias of DBM from leftover milk after feeding preparation, which may have affected our macronutrient analysis, but this poses new questions regarding human milk handling methods. Furthermore, while NIR human milk analyzers have been validated for precision in measuring protein and fat content, they are less accurate than mid-infrared analyzers [22,23]. A separate collaboration determined that the NIR analyzer used in this study may overestimate protein [24], suggesting that the protein content might be even lower than measured. Another limitation is the imprecision of length and head circumference measurements, and length boards had not been implemented as standard of care yet at the beginning of this study. We also sought to compare each infant’s daily protein and caloric intake with weekly growth velocities. However, despite a protocol designed to identify all unique pools purchased by the NICU as shipments arrived, some shipments were missed, preventing us from capturing 50/146 (34%) of the unique pools that were utilized in these infants. Because bottles of DBM from the same pool may be dispersed among multiple patients, this unfortunately precluded us from calculating the enteral nutrient intake for the majority of the patients.

## 5. Conclusions

Target-pooling DBM to meet a caloric minimum alone does not meet recommended protein intake for VLBW infants. Infants fed calorically target-pooled DBM still demonstrate a disproportionate negative change in length z-score over time and would likely benefit from more aggressive and earlier fortification strategies that target protein as well. Whether target-pooled DBM offers improved growth compared to random-pooled DBM remains unknown.

## Figures and Tables

**Table 1:** Infant characteristics. Mean ± SD or *n* (%).

	All Infants (*n* = 85)	Infants with 30 Days Growth Data (*n* = 69)	Infants Receiving <50% DBM (*n* = 27)	Infants Receiving >50% DBM (*n* = 42)	*p*-Value ^1^
Male (%)	47 (55.3%)	38 (55.1%)	17 (63.0%)	21 (50%)	0.291
Gestational Age (weeks)	29.4 ± 2.4	28.9 ± 2.0	29.6 ± 2.1	28.4 ± 1.8	0.011
Birth Weight (g)	1101.2 ± 266.5	1064.4 ± 260.0	1112.1 ± 264.5	1033.8 ± 255.5	0.225
SGA (%)	24 (28.2%)	11 (15.9%)	7 (25.9%)	4 (9.5%)	0.069
Days on DBM	26.5 ± 6.7	27.8 ± 5.4	24.9 ± 7.6	29.6 ± 1.7	<0.001
Infants on DBM at 30 days (%)	56 (65.9%) ^2^	50 (72.5%)	15 (55.6%)	35 (83.3%)	0.012
Infants Needing >24 kcal/oz (%)	59 (69.4%)	49 (71.0%)	14 (51.9%)	35 (83.3%)	0.005

^1^ For DBM subgroups; ^2^ 5 patients were switched from DBM to preterm formula at 27–28 days due to poor growth.

**Table 2:** Near-infrared (NIR) macronutrient analysis of donor breast milk (DBM) samples (*n* = 96).

	Calories (kcal/oz)	Protein (g/dL)	Fat (g/dL)	Carbohydrate (g/dL)
Minimum	12.43	0.26	1.48	6.29
Maximum	22.27	1.36	4.51	8.48
Mean	18.70	0.91	3.11	7.71
SD	1.75	0.19	0.57	0.38
Mean Coefficient of Variation	1.61%	6.81%	2.68%	1.81%

**Table 3:** Comparison of labeled and NIR measured caloric and protein concentrations.

	Calories (kcal/oz)			Protein (g/dL)		
	Label	NIR	Difference	Label	NIR	Difference
Minimum	19.51	12.43	−8.22	0.62	0.26	−0.62
Maximum	24.01	22.27	0.29	1.51	1.36	0.36
Mean	21.13	18.70	−2.43 ^1^	1.02	0.91	−0.11 ^1^
SD	1.01	1.75	1.66	0.18	0.19	0.21

^1^*p* < 0.001.

**Table 4:** Weekly growth velocities including subgroups by small for gestational age (SGA) status and DBM intake. Mean ± SD.

	Overall (*n* = 69)	SGA (*n* = 11)	AGA (*n* = 58)	*p*-Value	<50% DBM (*n* = 27)	>50% DBM (*n* = 42)	*p*-Value
Weight (g/kg/day)							
Week 1	8.84 ± 6.81	15.07 ± 9.42	7.66 ± 5.56	<0.001	8.50 ± 7.12	9.06 ± 6.68	0.742
Week 2	12.95 ± 5.75	17.00 ± 6.96	12.18 ± 5.21	<0.010	14.88 ± 6.08	11.71 ± 5.23	0.024
Week 3	16.58 ± 5.13	18.45 ± 5.91	16.23 ± 4.95	0.189	17.21 ± 3.38	16.17 ± 6.01	0.415
Week 4	16.11 ± 4.71	16.98 ± 3.71	15.94 ± 4.89	0.507	16.00 ± 3.50	16.18 ± 5.39	0.882
Length (cm/week)							
Week 1	0.59 ± 0.85	0.60 ± 0.87	0.59 ± 0.86	0.982	0.45 ± 0.81	0.68 ± 0.88	0.299
Week 2	0.97 ± 0.72	1.32 ± 0.72	0.91 ± 0.70	0.080	1.05 ± 0.65	0.92 ± 0.76	0.478
Week 3	0.95 ± 0.78	0.70 ± 0.96	1.00 ± 0.74	0.244	0.94 ± 0.85	0.96 ± 0.75	0.914
Week 4	1.03 ± 0.65	1.31 ± 0.45	0.97 ± 0.66	0.114	0.95 ± 0.67	1.08 ± 0.63	0.455
HC (cm/week)							
Week 1	0.14 ± 0.77	0.39 ± 0.91	0.09 ± 0.74	0.235	0.06 ± 0.73	0.19 ± 0.80	0.490
Week 2	0.67 ± 0.55	0.91 ± 0.58	0.63 ± 0.54	0.124	0.77 ± 0.58	0.61 ± 0.53	0.245
Week 3	1.01 ± 0.54	1.20 ± 0.42	0.98 ± 0.56	0.216	0.96 ± 0.55	1.05 ± 0.53	0.525
Week 4	1.01 ± 0.60	1.12 ± 0.59	0.99 ± 0.61	0.514	1.07 ± 0.59	0.97 ± 0.61	0.498

**Table 5:** z-scores of anthropometric measurements each week and at 36 weeks corrected gestational age (CGA). Mean ± SD.

	Weight	HC	Length	BMI
Birth	−0.52 ± 0.89	−0.45 ± 1.10	−0.51 ± 1.06	−0.30 ± 1.09
Week 1	−0.88 ± 0.81	−1.30 ± 0.96	−1.14 ± 1.06	−0.41 ± 0.95
Week 2	−1.04 ± 0.77	−1.45 ± 0.98	−1.28 ± 1.02	−0.43 ± 0.88
Week 3	−1.09 ± 0.79	−1.41 ± 0.95	−1.44 ± 1.05	−0.24 ± 0.82
Week 4	−1.13 ± 0.83	−1.28 ± 0.96	−1.60 ± 1.00	−0.09 ± 0.81
36 Weeks CGA	−1.03 ± 1.03	−0.68 ± 0.90	−1.64 ± 1.13	0.27 ± 0.85
Net Change	−0.51 ± 0.47	−0.23 ± 0.69	−1.01 ± 0.57	0.60 ± 0.93
